# Glutathione levels modulation as a strategy in host-parasite interactions—insights for biology of cancer

**DOI:** 10.3389/fphar.2014.00180

**Published:** 2014-08-05

**Authors:** Francesco Pennacchio, Antonio Masi, Alfonso Pompella

**Affiliations:** ^1^“E. Tremblay” Lab of Entomology, Department of Agriculture, “Federico II” University of NaplesNaples, Italy; ^2^Department of Agronomy, Food, Natural Resources, Animals and Environment, University of PaduaPadua, Italy; ^3^Redox Pathology Lab, Department of Translational Research NTMC, University of Pisa Medical SchoolPisa, Italy

**Keywords:** insect parasitism, bacterial infections, gamma-glutamyltransferase, exosomes, cancer progression

Parasitic organisms establish a symbiotic association with individuals of a different species—the host—to obtain the metabolic resources needed for their survival and reproduction. These antagonistic associations are characterized by a complex “host-parasite molecular cross-talk,” shaped by evolutionary processes largely driven by the requirement of making host's tissues more accessible to a successful colonization and exploitation (Hébert and Aubin-Horth, 2014). In a large number of cases, parasitism not only impairs host physiology and reproduction, but may even culminate with its death or complete consumption. This is the rule in insects, which include the largest number of species with parasitic life habits, characterized by subtle virulence strategies, often shared among organisms belonging to distant phylogenetic groups (Pennacchio and Strand, 2006). The astonishing diversity and the unexpected similarities shared with unrelated taxonomic entities offer the opportunity of insightful comparisons among virulence strategies targeting conserved molecular pathways, as a result of convergent evolutionary patterns.

Here we focus on a comparative analysis of a peculiar strategy of host physiological regulation, targeting the redox homeostasis, which appears to modulate parasitic interactions both at the organism and cellular level.

Insect studies, dealing with parasitism of aphids, have shown that the disruption of host GSH pool and metabolisms significantly contributes to its physiological regulation and castration (Pennacchio and Mancini, 2012). The parasitic wasp *Aphidius ervi* injects at the oviposition into host aphids a venom containing large amounts of a gamma-glutamyltransferase (*Ae-GGT)*, which triggers apoptosis in the upper part of the aphid ovarioles and therefore blocks oogenesis (Falabella et al., 2007). How this selective targeting is achieved and modulated at molecular level is still obscure; however, preliminary data indicate that in parasitized host aphid a depletion of GSH occurs, which primarily involves ovarian tissue (Masi and Pennacchio, unpublished results). It does not require a leap of imagination to speculate that injected *Ae*-GGT can compete with endogenous GGT for substrate GSH, thus interfering with the regular GSH cycle and exposing GSH-depleted cells to an oxidative stress, which ultimately triggers apoptosis. However, it is also reasonable to consider that the alteration of cell survival/apoptosis balance might ensue from the known pro-oxidant effects of GGT activity, due to increased metal-reducing ability of the GSH metabolite cysteinyl-glycine and extracellular production of reactive oxygen species (Paolicchi et al., 2002). The presence of a GGT in the genome of an entomopoxvirus associated with the parasitic wasp *Diachasmimorpha longicaudata* (Hashimoto and Lawrence, 2005), injected at the oviposition and used as a delivery system of virulence factors, indicates that this enzyme may have additional regulatory roles of host physiology, which remain largely elusive. The possible negative impact on host immunity (Clark et al., 2010) is certainly worth of consideration.

Studies on virulence factors of microrganisms have documented that the invasion strategies of selected pathogenic bacteria also target host GSH metabolism. Indeed, it has been shown that GGT activity of *Helicobacter pylori* and *H. suis*, the agents responsible of peptic ulcer, can exert antiproliferative and pro-apoptotic effects in gastric epithelial cells (Schmees et al., 2007; Gong et al., 2010; Ricci et al., 2014). These effects are triggered by GGT-dependent metabolism of GSH and production of H_2_O_2_, which result in the activation of NF-kB, up-regulation of interleukin-8 and increased oxidative DNA damage (Gong et al., 2010; Ricci et al., 2014). Paradoxically enough, supplementation with glutathione of *H. pylori* GGT-treated cells strongly enhanced the harmful effects (Flahou et al., 2011; Zhang et al., 2013). By confocal microscopy, *H. suis* outer membrane vescicles (OMV)—submicroscopic structures 20–50 nm in diamater, budding from the cell surface—were identified as carriers of *H. suis* GGT, capable of delivering the enzyme to the deeper mucosal layers (Zhang et al., 2013). In association with such membranous structures, active GGT from *H. suis* in fact translocates across the epithelial layers and can access lymphocytes residing in the lamina propria of gastric mucosa. The result of this intriguing process appears to be an inhibition of lymphocyte proliferation, i.e., a perturbation of host immunity and a facilitation of bacterial infection (Oertli et al., 2013). Future studies will likely expand the number of examples of GSH-based host-parasite interactions, as possibly in the case of pro-apoptotic activity of GGT released by *Campylobacter jejunii* (Barnes et al., 2007). Cellular GSH appears, thus, to represent a conserved target for parasitic (micro)organisms which aim at altering host redox homeostasis to weaken its immune defenses, using GGT as a key-element of a virulence strategy.

It is possible to further elaborate on this concept by jumping in the field of cancer biology and taking into account the “parasitic” behavior exhibited by malignant cells spreading across tissues and organs of the patient (the “host”). GGT activity is in fact expressed in a number of malignant tumors, and expression levels often increase along with progression of the disease and appearance of more invasive phenotypes (reviewed in Pompella et al., 2006). Importantly, recent studies showed that active GGT can be released from cells, including cancer cells (Franzini et al., 2009), in association with submicroscopic vesicles, 20–40 nm in diameter, resembling exosomes (Fornaciari et al., 2014). The similarity of such structures with GGT-rich OMV particles of *H. pylori* and *H. suis* is indeed obvious. GGT activity of cancer cells can affect intracellular redox equilibria (Pompella et al., 2007), and produces in addition significant extracellular effects, on the S-thiolation status of extracellular proteins (Corti et al., 2005), as well as on the redox status and ligand binding affinity of cell surface receptors related with cell survival/apoptosis balance (Dominici et al., 2004). The question therefore arises, whether GGT-rich exosomes shed by cancer cells can produce in host's surrounding tissues effects comparable to those reported for *Ae*-GGT or *Helicobacter* GGT, possibly resulting in facilitation of malignant cells survival and diffusion.

Collectively, these GSH/GGT-dependent processes described in evolutionary distant organisms and different cell populations further corroborate the pivotal importance of redox homeostasis in the modulation of health and disease conditions; shedding light on regulatory elements of these convergent parasitic strategies will likely allow the identification of new potential targets for therapy.

## Conflict of interest statement

The authors declare that the research was conducted in the absence of any commercial or financial relationships that could be construed as a potential conflict of interest.
